# The cumulative impact of type 2 diabetes and obstructive sleep apnoea on cardiovascular, liver, diabetes‐related and cancer outcomes

**DOI:** 10.1111/dom.16059

**Published:** 2024-11-11

**Authors:** David R. Riley, Alex Henney, Matthew Anson, Gema Hernadez, Sizheng S. Zhao, Uazman Alam, John P. H. Wilding, Sonya Craig, Daniel J. Cuthbertson

**Affiliations:** ^1^ Department of Cardiovascular and Metabolic Medicine University of Liverpool Liverpool UK; ^2^ Department of Diabetes, Obesity and Endocrinology University Hospital Aintree, Liverpool University NHS Foundation Trust Liverpool UK; ^3^ Liverpool Centre for Cardiovascular Science at University of Liverpool, Liverpool John Moores University and Liverpool Heart & Chest Hospital Liverpool UK; ^4^ TriNetX LLC Cambridge Massachusetts USA; ^5^ Centre for Musculoskeletal Research at University of Manchester Manchester UK; ^6^ Liverpool Sleep & Ventilation Unit Aintree University Hospitals NHS Foundation Trust Liverpool UK

**Keywords:** cardiovascular disease, dementia, malignancy, microvascular complications, obstructive sleep apnoea, type 2 diabetes

## Abstract

**Aim:**

A bidirectional relationship exists between obstructive sleep apnoea (OSA) and type 2 diabetes (T2D). We aimed to examine the cumulative impact of having both OSA and T2D on patient outcomes, relative to having either condition alone.

**Materials and methods:**

Using TriNetX, a global federated research network (*n* = 128 million), we undertook two retrospective cohort studies, using time‐to‐event analysis. Analysis 1 compared OSA with T2D versus OSA alone; analysis 2 compared T2D with OSA versus T2D alone. Propensity score matching using greedy nearest neighbour (calliper 0.1) balanced the cohorts (1:1) for significant covariates. Primary outcomes were cardiovascular, liver, diabetes‐related (microvascular) and cancer events over 1–5 years.

**Results:**

Analysis 1 (*n* = 179 688): A codiagnosis of T2D/OSA significantly increased risk of all‐cause mortality (hazard ratio [HR] 1.52; confidence interval [CI]: 1.48, 1.57), dementia (HR 1.19; CI: 1.12, 1.26), liver (HR 2.20; CI: 1.77, 2.73), pancreatic (HR 1.62; CI: 1.35, 1.93), colon, renal and endometrial cancers; all cardiovascular, microvascular and liver related outcomes versus OSA alone over 1–5 5 years following OSA diagnosis. Analysis 2 (*n* = 240 094): A codiagnosis of OSA/T2D significantly increased the risk of peripheral (HR 1.39; CI: 1.36, 1.43) and autonomic (HR 1.63; CI: 1.51, 1.75) neuropathy; retinopathy (HR 1.13; CI: 1.09, 1.18), CKD (HR 1.21; CI: 1.18, 1.23); all cardiovascular and liver outcomes; all‐cause mortality and several obesity related cancers versus T2D alone.

**Conclusions:**

T2D significantly potentiates risk of cardiovascular, malignancy and liver‐related outcomes in individuals with OSA. OSA, in individuals with T2D, significantly potentiates risk of cardiovascular disease, malignancy, death and several microvascular complications (retinopathy, CKD, peripheral/autonomic neuropathy).

## 1 INTRODUCTION

The obesity pandemic has driven an adiposopathy epidemic, with a rapid rise in incidence of adiposity‐related complications including cardiovascular disease (CVD), metabolic dysfunction–associated steatotic liver disease (MASLD), type 2 diabetes (T2D) and obstructive sleep apnoea (OSA).^1^ This clustering of such complications is associated with significant morbidity, mortality and impaired quality of life.^2^ The cooccurrence of T2D and OSA suggests a bidirectional relationship likely exists independent of BMI or waist circumference as surrogates for adiposity,^3^ posing a distinct challenge. This is biologically plausible given features of OSA (intermittent hypoxaemia, autonomic nervous system [ANS] overactivity and sleep fragmentation) promote elevated systemic inflammation, insulin resistance, glucose intolerance and β‐cell dysfunction, whilst, conversely, features of T2D (diabetic neuropathy, insulin resistance, chronic inflammation and oxidative stress) promote sleep‐disordered breathing through ANS and ventilatory control dysfunction^4^; however, as these studies did not directly measure adiposity or fat distribution, it remains possible associations are purely related to differences in these measures between individuals.

This bidirectional relationship is crucial given the individual risks inherent with both T2D and OSA. T2D is associated with major adverse cardiovascular events (MACE), heart failure, major adverse liver outcomes (MALO) and site‐specific cancers.^5^ Previously, MACE was the leading cause of death in patients with T2D^6^; however, over the last decade, cancer mortality, and specifically, colorectal, hepatocellular, gallbladder, breast, endometrial and pancreatic cancers, has surpassed MACE.^7^, ^8^ Similarly, OSA amplifies CVD risk,^9^ with the American Heart Association advocating CVD risk reduction in patients with OSA.^10^ Moreover, OSA is associated with more aggressive MASLD,^11^ whilst also increasing risk of separate site‐specific cancers to T2D.^12^

Clinical trials have shown effective cardiovascular risk reduction with lipid‐lowering therapy, antihypertensive, sodium‐glucose cotransporter‐2 inhibitors (SGLT2i) and novel incretin–based glucose‐lowering therapy has reduced mortality, incidence of CVD, including heart failure^13^, ^14^ and microvascular complications in patients with T2D.^15^ However, given the global prevalence and increasing incidence of T2D, the burden of these complications remains challenging on a population level. Therefore, identifying specific at‐risk groups to target preventive strategies towards is crucial. Patients with concurrent T2D and OSA may represent one such high‐risk phenotype, although current literature has not adequately defined the extent to which the cooccurrence of T2D and OSA impacts on CVD, MASLD, diabetes‐related complications and cancer, beyond either pathology in isolation. It is plausible that multiple, distinct mechanistic pathways are implicated that may potentiate risk of these complications, and these patients warrant targeted screening.

The aims of this study were to assess whether a cumulative impact exists on cardiovascular, liver, diabetes‐related and cancer outcomes, by having a co‐diagnosis of T2D and OSA.

## 2 MATERIALS AND METHODS

### 2.1 Study populations

We performed a large retrospective cohort study using the TriNetX dataset. TriNetX is a global federated research network with access to inpatient and outpatient health‐related data, including diagnoses, procedures, medications and laboratory values. Using their Global Collaborative Network, we can access the anonymised health data of 128 million patients aged 18 or over, from 17 different countries, from predominantly US healthcare organizations (HCOs). To assimilate data from HCOs that join the network, data are mapped to a common data model to reflect individual institution, country and regional standards regarding electronic health record data. All data collection, processing and transmission are performed in compliance with all Data Protection laws applicable to the contributing HCOs, including the EU Data Protection Law Regulation 2016/679, the General Data Protection Regulation on the protection of natural persons regarding the processing of personal data and the Health Insurance Portability and Accountability Act, the US federal law which protects the privacy and security of healthcare data. The TriNetX Global Collaborative Network is a distributed network (with most HCOs located in the USA), and analytics are performed at the HCO with only aggregate results being surfaced and returned to the platform. Data usage and publication agreements are in place with all HCOs. Data for this paper were extracted from TriNetX on the 21st of June 2024. Further information on the network has been described in detail elsewhere.^16^

### 2.2 Building cohorts in TriNetX

#### 2.2.1 Analysis 1: Comparison of OSA with T2D versus OSA alone (OSA + T2D vs. OSA)

Adult patients (age ≥ 18 years) diagnosed with OSA were identified using the ICD‐10 CM: G47.33 code; additionally, diagnosis must have been at least 5 years before the date of data extraction. Patients were then split into two cohorts based on their T2D status, ICD‐10 CM: E11. In the OSA + T2D cohort, patients were included if they had their first coding of T2D within 12 months of OSA diagnosis. Patients with type 1 diabetes (ICD‐10 CM: E10) are excluded from this cohort. In the OSA only cohort, patients were excluded if they had a diagnosis of diabetes, ICD‐10 CM: E08‐E013. The two cohorts were propensity score matched (PSM) 1:1, using greedy nearest neighbour matching and a calliper of 0.1, for age at index event, sex, ethnicity (white, black or African American), prior diagnoses of hypertension, ischaemic heart disease (IHD), dyslipidaemia, chronic kidney disease (CKD) and body mass index (BMI). Covariates were deemed well matched if the standardized mean difference (SMD) was <0.1. The participants in this analysis were also matched on continuous positive airway pressure (CPAP) initiation. Importantly, not all patients had BMI data available at baseline, as such, a separate sensitivity analysis was also undertaken to ensure this had no significant impact on the study findings.

#### 2.2.2 Analysis 2: Comparison of T2D with OSA versus T2D alone (T2D + OSA vs. T2D)

Adult patients (age ≥ 18 years) diagnosed with T2D were identified using the ICD‐10 CM: E11 code; additionally, the diagnosis must have been at least 5 years before the date of data extraction. Patients with type 1 diabetes were excluded using the ICD‐10 CM: E10 code. Patients were then split into two cohorts based on their OSA status, ICD‐10 CM: G47.33. In the T2D + OSA cohort, patients were included if their first coding of OSA was within 12 months of T2D diagnosis. In the T2D, only cohort patients were excluded if they had a diagnosis of OSA. PSM for this analysis was the same as analysis one, except CPAP is replaced with HbA1c. Like BMI, not all patients had baseline HbA1c data available, so a sensitivity analysis was undertaken where both cohorts had 100% BMI and HbA1c data available to ensure this absence of data had no significant impact on the study findings.

### 2.3 Outcomes

Time to event analysis, using Cox proportional hazards models, was performed over a 1‐to‐5‐year period from the index event. Outcomes were not collected in the first 12 months after the index event as this would introduce an immortal time bias in favour of the combined cohorts (analysis one: OSA + T2D and analysis two: T2D + OSA). Outcome data were included from 366 to 1825 days after the index event. The primary outcomes were split into three main groups: cardiovascular events (CVE) (IHD, ischaemic stroke, heart failure and atrial fibrillation/flutter); cancer (hepatocellular, pancreatic, breast, colon, cholangiocarcinoma, renal, oesophageal and endometrial); microvascular complications (peripheral neuropathy, macular oedema, retinopathy, amputation, autonomic neuropathy, CKD and foot ulcers). Additional outcomes include all‐cause mortality, dementia, MASLD and metabolic dysfunction‐associated steatohepatitis (MASH). All data were collected directly from electronic medical records; no data were collected from government sources or registered persons databases, so mortality data may be underreported, see Table S1 for a full list of codes used for each outcome.

### 2.4 Identification of the index event and the time window

The index event defines the point at which outcome data can start to be collected, day 1. In analysis 1, the index event was OSA diagnosis, and in analysis 2, T2D diagnosis. In both analyses, outcome data were included from days 366 to 1825 after the index event. The results of a sensitivity analysis using data from days 1 to 1825 can be found in Tables S2 and S3. The choice to only use data from days 366 to 1825 was done to prevent an immortal time bias in favour of the combined cohorts, OSA + T2D in analysis 1 and T2D + OSA in analysis 2.

### 2.5 Sensitivity analyses

Several sensitivity analyses were completed and are included as supplemental information. These sensitivity analyses explored the effect of including the first year in the analyses, the impact of the absent BMI and HbA1c data at baseline and the effect of excluding patients initiated on CPAP in analysis one and excluding patients initiated on SGLT2i, glucagon‐like peptide‐1 receptor agonists (GLP‐1RA) and other incretin‐based therapies in analysis 2 (Tables [Link], [Link]).

### 2.6 Statistical analysis

Cox regression analyses were performed using the TriNetX platform for each outcome. These analyses are unadjusted for other covariates as the platform does not allow for the generation of adjusted Cox regression models. Patients who had experienced an outcome prior to the index event were excluded from that specific outcome analysis. Data were collected from 12 months after the date of the index event till 5 years after and was used to generate a hazard ratio (HR) with 95% confidence interval (CI), log rank test and Kaplan–Meier's survival curve. Censoring was applied to patients when there was no more data available. A patient was removed (censored) from the analysis after the last event in their electronic record. TriNetX uses the R's Survival package v3.2‐3 for survival analyses.

## 3 RESULTS

### 3.1 Propensity score matching and follow‐up

Prior to PSM in analysis one (OSA + T2D vs. OSA), there were 180 129 patients in the combined cohort and 1 126 548 patients in the OSA only cohort. The greatest differences in covariates were seen in age (SMD 0.538), IHD (SMD 0.410), CKD (SMD 0.369), hypertension (SMD 0.476), lipid disorders (SMD 0.376) and BMI (SMD 0.516). Following PSM, all covariates had a SMD <0.1, and were deemed well matched; cohort sizes were now matched at 179 688 patients. Median follow‐up (days) after PSM was 1775 (IQR 1349) for the combined cohort and 1825 (IQR 1019) in the OSA only group (Table 1).

**TABLE 1:** Propensity score matching.

Analysis 1: OSA + T2D vs. OSA							
Covariate		Prepropensity score matching			Postpropensity score matching		
		OSA + T2D	OSA	SMD	OSA + T2D	OSA	SMD
Sample size^a^		180 129	1 126 548		179 688	179 688	
Age at index event—Mean ± SD (years)		60.0 ± 12.8	51.5 ± 18.2	0.538	60.0 ± 12.8	60.1 ± 13.2	0.007
Sex (%)	Female	38.7	39.8	0.022	38.7	38.9	0.004
	Male	56.7	56.4	0.005	56.7	56.3	0.008
Ethnicity—White (%)		64.5	68.0	0.075	64.5	64.6	0.003
Ethnicity—Black or African American (%)		13.2	10.1	0.095	13.2	12.9	0.009
Hypertension^a^		113 908 (63.4%)	452 387 (40.2%)	0.476	113 907 (63.4%)	119 950 (66.8%)	0.071
IHD^a^		49 646 (27.6%)	131 237 (11.7%)	0.410	49 645 (27.6%)	48 241 (26.8%)	0.018
CKD^a^		27 167 (15.1%)	48 925 (4.4%)	0.369	27 166 (15.1%)	24 471 (13.6%)	0.043
Dyslipidaemia^a^		91 681 (51.0%)	368 680 (32.8%)	0.376	91 680 (51.0%)	92 121 (51.3%)	0.005
BMI—Mean ± SD (kg/m^2^)		38.2 ± 8.9	33.7 ± 8.5	0.516	38.2 ± 8.9	37.9 ± 8.6	0.033
CPAP^a^		6392 (3.6%)	15 674 (1.4%)	0.140	6391 (3.6%)	5772 (3.2%)	0.019

Analysis 2: T2D + OSA versus T2D							
		Prepropensity score matching			Postpropensity score matching		
Covariate		T2D + OSA	T2D	SMD	T2D + OSA	T2D	SMD
Sample size^a^		240 533	4 829 108		240 094	240 094	
Age at index event—Mean ± SD (yrs)		60.3 ± 12.6	61.4 ± 14.6	0.084	60.3 ± 12.6	60.3 ± 12.6	0.001
Sex (%)	Female	40.0	48.6	0.173	40.0	40.0	<0.001
	Male	55.3	48.7	0.133	55.3	55.3	<0.001
Ethnicity—White		64.1	54.8	0.190	64.1	64.1	<0.001
Ethnicity—Black or African American		14.4	14.2	0.005	14.4	14.4	<0.001
Hypertension^a^		153 939 (64.1%)	2 558 622 (55.5%)	0.177	153 939 (64.1%)	154 005 (64.1%)	0.001
IHD^a^		61 921 (25.8%)	839 897 (18.2%)	0.184	61 921 (25.8%)	61 849 (25.8%)	0.001
CKD^a^		34 839 (14.5%)	439 795 (9.5%)	0.153	34 839 (14.5%)	34 898 (14.5%)	0.001
Dyslipidaemia^a^		120 486 (50.2%)	1 820 215 (39.5%)	0.217	153 939 (64.1%)	154 005 (64.1%)	<0.001
BMI—Mean ± SD (kg/m^2^)		37.9 ± 8.8	32.2 ± 8.1	0.674	37.9 ± 8.8	37.4 ± 8.3	0.063
HbA1c—Mean ± SD (%)		7.2 ± 1.9	7.3 ± 2.0	0.022	7.2 ± 1.9	7.3 ± 2.0	0.035

Abbreviations: BMI, body mass index; CKD, chronic kidney disease; CPAP, continuous positive airway pressure; IHD, ischaemic heart disease; OSA, obstructive sleep apnoea; SD, standard deviation; SMD, standardized mean difference; T2D, type 2 diabetes.

^a^ Number of participants.

**FIGURE 1:**
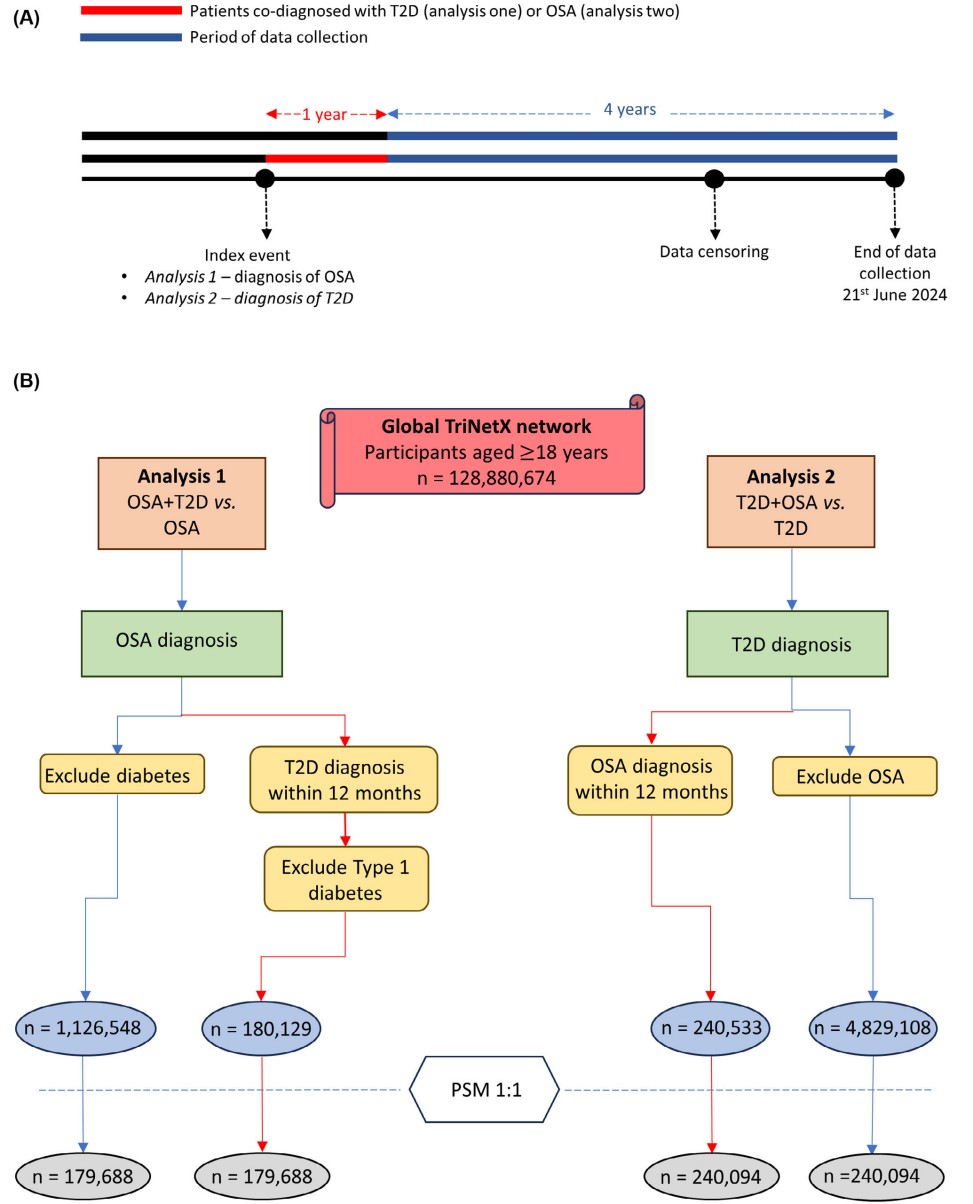
Study overview. (A) A timeline showing the period of data collection and how it relates to diagnoses of T2D and OSA. (B) A schematic demonstrating how the cohorts were created for the two analyses. OSA, obstructive sleep apnoea; T2D, type 2 diabetes.

Prior to PSM in analysis 2 (T2D + OSA vs. T2D), there were 240 533 patients in the T2D + OSA cohort and 4 829 108 patients in the T2D only cohort. The greatest difference in covariate was seen in BMI (SMD 0.674). Following PSM, all covariates had a SMD <0.1; cohort sizes were now matched at 240 094. Median follow‐up (days) after PSM was 1818 (IQR 1249) in the T2D + OSA cohort and 1825 (IQR 1296) in the T2D only cohort (Figure 1).

### 3.2 Log rank test

For analysis 1 (OSA + T2D vs. OSA), the log rank test demonstrated a significant difference (*p* < 0.05) in the survival curves for all cardiovascular, microvascular, dementia, all‐cause mortality and steatotic liver disease–related outcomes. In cancer outcomes, there was a significant difference in endometrial, renal, colon, pancreatic and hepatocellular cancers with no significant difference in breast or cholangiocarcinoma. In analyses 2 (T2D + OSA vs. T2D), there was a significant difference in the survival curves for all cardiovascular outcomes, dementia, all‐cause mortality and steatotic liver disease–related outcomes. In microvascular and cancer outcomes, there was a significant difference in the survival curves for peripheral neuropathy, retinopathy, autonomic neuropathy, CKD, foot ulcers, breast and renal cancers. There was no significant difference in the remaining microvascular and neoplastic outcomes. See Table 2A,B for the complete log rank test results for analyses 1 and 2, respectively.

**TABLE 2:** Summary of outcome incidence, survival probability and log rank test for analysis 1 (OSA + T2D vs. OSA) and analysis 2 (T2D + OSA vs. T2D).

	Analysis 1 (OSA + T2D vs. OSA)						Analysis 2 (T2D + OSA vs. T2D)					
	Cohorts	Sample size^a^	Outcome	5‐year survival probability	Log rank test	*p* value	Cohorts	Sample size^a^	Outcome	5‐year survival probability	Log rank test	*p* value
Peripheral neuropathy	OSA + T2D	159 220	11 424	88.91%	4756.417	<0.001	T2D + OSA	206 555	17 582	87.25%	851.726	<0.001
	OSA	173 915	4440	96.34%			T2D	219 922	13 636	90.60%		
Macular oedema	OSA + T2D	178 340	953	99.17%	690.635	<0.001	T2D + OSA	237 255	1936	98.77%	0.336	0.562
	OSA	179 408	141	99.89%			T2D	237 011	1969	98.75%		
Retinopathy (excluding macular oedema)	OSA + T2D	175 744	2786	97.52%	1849.826	<0.001	T2D + OSA	232 377	5262	96.57%	38.752	<0.001
	OSA	178 768	516	99.59%			T2D	233 305	4671	96.96%		
Amputations	OSA + T2D	179 247	335	99.70%	171.261	<0.001	T2D + OSA	239 324	574	99.63%	0.013	0.909
	OSA	179 539	87	99.93%			T2D	239 278	579	99.63%		
Autonomic neuropathy	OSA + T2D	178 474	1174	98.96%	1206.978	<0.001	T2D + OSA	237 806	1956	98.74%	179.902	<0.001
	OSA	179 650	32	99.97%			T2D	238 852	1212	99.23%		
CKD	OSA + T2D	143 756	10 792	88.85%	1680.037	<0.001	T2D + OSA	188 323	16 381	87.47%	268.630	<0.001
	OSA	151 987	6693	93.87%			T2D	196 462	14 188	89.41%		
Foot ulcers	OSA + T2D	175 287	3215	97.13%	2038.687	<0.001	T2D + OSA	232 493	5264	96.58%	74.880	<0.001
	OSA	178 894	648	99.48%			T2D	233 635	4449	97.11%		

Cardiovascular outcomes	Cohorts	Sample size^a^	Outcome	5‐year survival probability	Log rank test	*p* value	Cohorts	Sample size^a^	Outcome	5‐year survival probability	Log rank test	*p* value
Ischaemic heart disease	OSA + T2D	119 135	11 361	85.97%	884.953	<0.001	T2D + OSA	156 775	16 572	84.88%	311.632	<0.001
	OSA	126 465	8684	90.51%			T2D	167 389	14 526	87.39%		
Heart failure	OSA + T2D	135 033	10 093	89.08%	918.152	<0.001	T2D + OSA	179 064	14 604	88.43%	405.814	<0.001
	OSA	151 270	7815	92.90%			T2D	203 909	12 906	90.76%		
Atrial fibrillation	OSA + T2D	144 102	7169	92.57%	186.632	<0.001	T2D + OSA	193 088	10 409	92.15%	155.220	<0.001
	OSA	148 903	6366	94.04%			T2D	210 877	9437	93.35%		
Ischaemic stroke	OSA + T2D	170 287	3700	96.59%	203.670	<0.001	T2D + OSA	226 610	5640	96.21%	42.181	<0.001
	OSA	172 303	2900	97.59%			T2D	229 170	5048	96.65%		

Neoplastic outcomes	Cohorts	Sample size^a^	Outcome	5‐year survival probability	Log rank test	*p* value	Cohorts	Sample size^a^	Outcome	5‐year survival probability	Log rank test	*p* value
Liver cancer	OSA + T2D	179 126	248	99.78%	54.115	<0.001	T2D + OSA	239 254	401	99.74%	0.070	0.791
	OSA	179 391	124	99.90%			T2D	239 190	394	99.75%		
Pancreatic cancer	OSA + T2D	178 804	298	99.74%	28.315	<0.001	T2D + OSA	238 891	411	99.74%	0.432	0.511
	OSA	179 338	203	99.84%			T2D	239 018	431	99.72%		
Breast cancer	OSA + T2D	177 274	861	99.24%	0.219	0.640	T2D + OSA	236 677	1294	99.16%	7.082	0.008
	OSA	176 739	922	99.25%			T2D	236 899	1165	99.24%		
Colon cancer	OSA + T2D	178 427	501	99.56%	15.550	<0.001	T2D + OSA	238 275	747	99.52%	0.096	0.756
	OSA	178 473	425	99.66%			T2D	238 350	760	99.51%		
Cholangiocarcinoma	OSA + T2D	179 650	25	99.98%	1.853	0.173	T2D + OSA	240 034	30	99.98%	0.544	0.461
	OSA	179 643	18	99.99%			T2D	240 028	36	99.98%		
Renal cancer	OSA + T2D	178 197	510	99.55%	5.025	0.025	T2D + OSA	237 928	768	99.51%	8.075	0.004
	OSA	178 168	486	99.61%			T2D	238 498	663	99.58%		
Oesophageal cancer	OSA + T2D	179 323	128	99.89%	2.459	0.117	T2D + OSA	239 544	186	99.88%	0.567	0.451
	OSA	179 391	115	99.91%			T2D	239 680	172	99.89%		
Endometrial cancer	OSA + T2D	178 757	284	99.75%	10.159	0.001	T2D + OSA	238 740	402	99.74%	0.075	0.784
	OSA	178 894	236	99.81%			T2D	238 872	395	99.74%		

All‐cause mortality, dementia and liver outcomes	Cohorts	Sample size^a^	Outcome	5‐year survival probability	Log rank test	*p* value	Cohorts	Sample size^a^	Outcome	5‐year survival probability	Log rank test	*p* value
All‐cause mortality	OSA + T2D	179 688	14 841	87.73%	1013.070	<0.001	T2D + OSA	240 094	21 751	86.92%	296.774	<0.001
	OSA	179 688	10 977	91.57%			T2D	240 094	18 376	88.84%		
Dementia	OSA + T2D	175 351	2524	97.76%	35.657	<0.001	T2D + OSA	233 954	3854	97.50%	45.036	<0.001
	OSA	175 912	2343	98.11%			T2D	234 870	3312	97.85%		
Metabolic dysfunction‐associated steatotic liver disease	OSA + T2D	169 014	6435	94.01%	719.643	<0.001	T2D + OSA	225 473	9122	93.83%	467.791	<0.001
	OSA	172 631	4366	96.37%			T2D	230 440	6667	95.56%		
Metabolic dysfunction‐associated steatohepatitis	OSA + T2D	177 528	1492	98.68%	488.367	<0.001	T2D + OSA	236 912	2137	98.63%	150.173	<0.001
	OSA	178 839	594	99.52%			T2D	238 383	1422	99.09%		

Abbreviations: CKD, chronic kidney disease; OSA, obstructive sleep apnoea; T2D: type 2 diabetes.

^a^ Number of participants.

### 3.3 Analysis 1: The impact of T2D on outcomes for individuals with OSA (OSA + T2D vs. OSA) from 12 months to 5 years post‐OSA diagnosis

#### 3.3.1 Cardiovascular events (Figure 2A)

**FIGURE 2:**
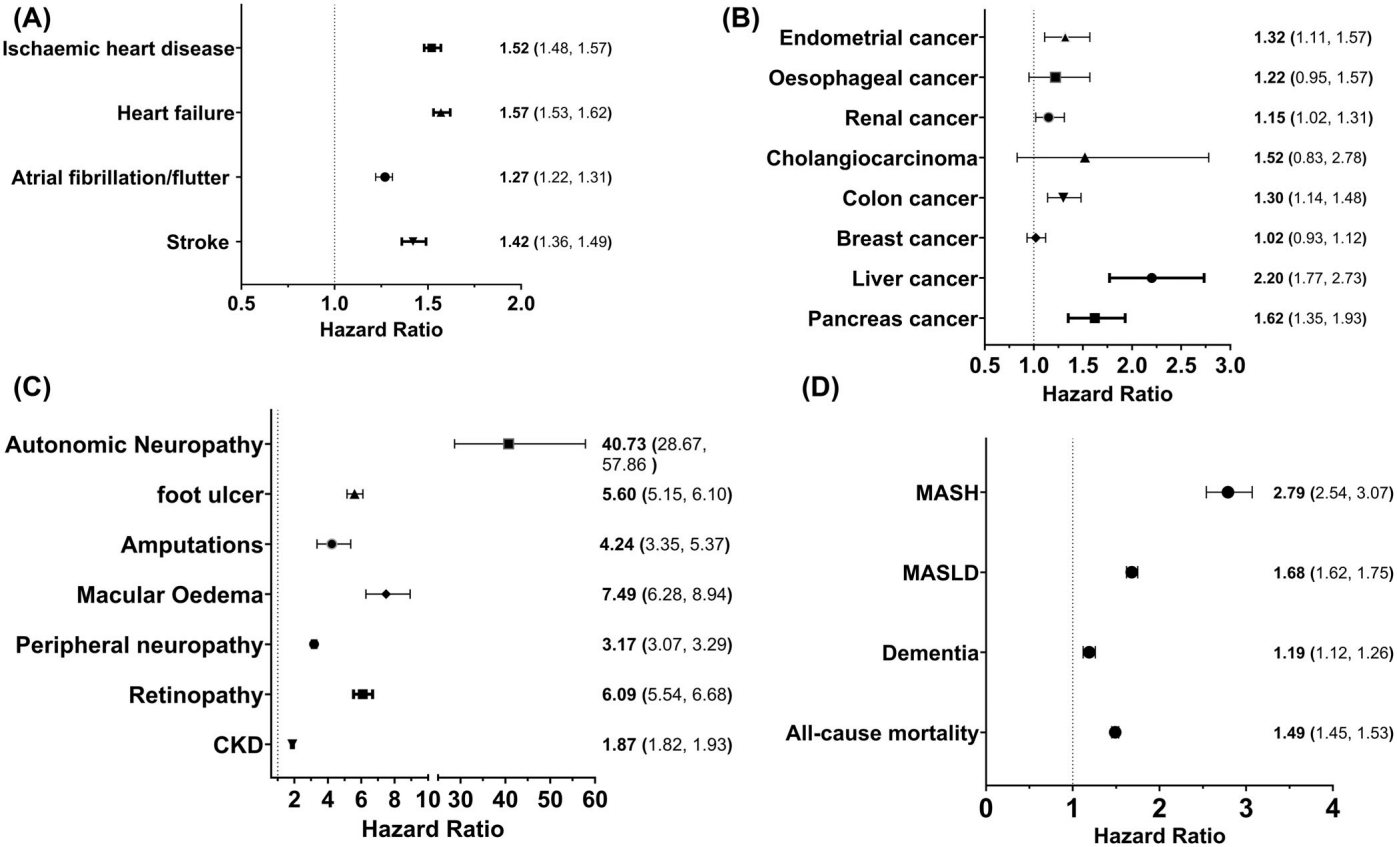
Hazard ratios of people with OSA and T2D versus OSA over a 1‐ to 5‐year period from OSA diagnosis: (A) cardiovascular events, (B) malignancy, (C) microvascular complications, and (D) all‐cause mortality, dementia and fatty liver disease. CKD, chronic kidney disease; MASH, metabolic dysfunction–associated steatohepatitis; MASLD, metabolic dysfunction–associated steatotic liver disease; OSA, obstructive sleep apnoea; T2D, type 2 diabetes.

The development of T2D within a year of OSA diagnosis was associated with a significant increased risk for all CVE when compared to a T2D diabetes naive cohort. The largest increase in risk was seen in the development of heart failure (HR 1.57 CI: 1.53, 1.62).

#### 3.3.2 Cancer diagnoses (Figure 2B)

A codiagnosis of T2D in individuals with OSA was associated with a significant increased risk of endometrial, renal, colon, pancreatic and hepatocellular cancers with the greatest increase in risk seen with hepatocellular cancer (HR 2.20 CI: 1.77, 2.73). There was no significant increase in the rate of breast, cholangiocarcinoma or oesophageal cancers.

#### 3.3.3 Microvascular complications (Figure 2C)

The presence of T2D in individuals with OSA significantly increased the risk of all microvascular outcomes with the greatest increase in risk seen with autonomic neuropathy (HR 40.73 CI: 28.67, 57.86).

#### 3.3.4 All‐cause mortality, dementia and fatty liver disease (Figure 2D)

The development of T2D within a year of OSA diagnosis was associated with a significant increased risk of all‐cause mortality (HR 1.49 CI: 1.45, 1.53), dementia (HR 1.19 CI: 1.12, 1.26) and steatotic liver disease.

### 3.4 Analysis 2: The impact of OSA on outcomes for individuals with T2D (T2D + OSA vs. T2D) from 12 months to 5 years postdiabetes diagnosis

#### 3.4.1 Cardiovascular events (Figure 3A)

**FIGURE 3:**
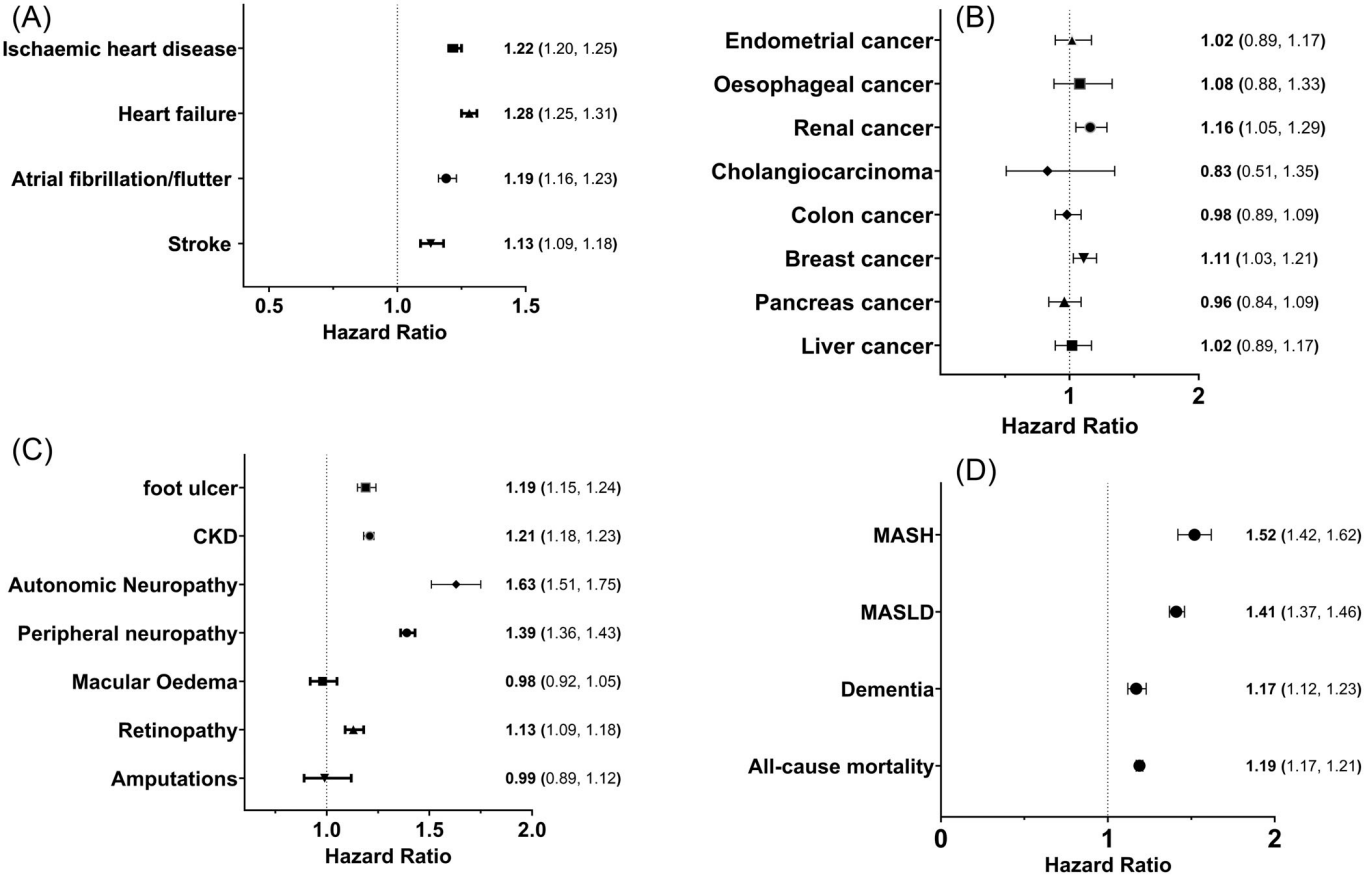
Hazard ratios of people with T2D and OSA versus T2D over a 1‐ to 5‐year period from T2D diagnosis: (A) cardiovascular events, (B) malignancy, (C) microvascular complications, and (D) All‐cause mortality, dementia, and fatty liver disease. CKD, chronic kidney disease; MASH, metabolic dysfunction–associated steatohepatitis; MASLD, metabolic dysfunction–associated steatotic liver disease; OSA, obstructive sleep apnoea; T2D, type 2 diabetes.

The development of OSA within a year of T2D diagnosis was associated with a significant increased risk for all CVE when compared to a OSA naive cohort. The largest increase in risk was seen in the development of heart failure (HR 1.28 CI: 1.25, 1.31).

#### 3.4.2 Cancer diagnosis (Figure 3B)

A codiagnosis of OSA in individuals with T2D was associated with a significant increased risk of breast (HR 1.11 CI: 1.03, 1.21) and renal (HR 1.16 CI: 1.05, 1.29) cancer. There was no significant increase in the rate of endometrial, cholangiocarcinoma, colon, pancreatic, hepatocellular or oesophageal cancer.

#### 3.4.3 Microvascular complications (Figure 3C)

The presence of OSA in individuals with T2D significantly increased the risk of peripheral neuropathy, retinopathy, autonomic neuropathy, CKD and foot ulcer formation. The greatest increase in risk was seen with autonomic neuropathy (HR 1.63. CI: 1.51, 1.75). There was no significant change in the risk of macular oedema or amputation.

#### 3.4.4 All‐cause mortality, dementia and fatty liver disease (Figure 3D)

The development of OSA within a year of T2D diagnosis was associated with a significant increased risk of all‐cause mortality (HR 1.19 CI: 1.17, 1.21), dementia (HR 1.17 CI: 1.12, 1.23) and fatty liver disease.

## 4 DISCUSSION

In this large retrospective real‐world study, we explored the impact of a codiagnosis of T2D and OSA on incident rates of CVE, obesity‐related malignancy, microvascular complications, MASLD, dementia and all‐cause mortality. We robustly demonstrate the strong cumulative impact of these conditions in amplification of risk for several significant clinical outcomes: CVE (IHD, heart failure, ischaemic stroke and AF), MASLD, dementia, microvascular complications, certain obesity related cancers and thus all‐cause mortality over a 4‐year period, when compared to each condition independently. Tentatively, our data also suggest a greater impact on the development of T2D in individuals with OSA on cardiovascular, microvascular and liver related outcomes compared to those with T2D who develop OSA supporting the notion that aggressive weight loss management in individuals with OSA may provide a greater health economic benefit through preventing the development of T2D. However, this finding could be due to variation in visceral and ectopic fat distribution between the cohorts and is not something controlled for in this study^17^; this finding would need confirmation in prospective studies.

The increased risk of CVE and all‐cause mortality with both diagnoses is consistent with current literature.^18^, ^19^, ^20^ With respect to microvascular complications, unsurprisingly, our data support the well‐established paradigm that development of T2D increases the risk of these complications. However, in analysis 2 (T2D + OSA vs. T2D), we establish that addition of a diagnosis of OSA in individuals with T2D significantly impacts the rate these complications develop, above that seen in individuals with only T2D, specifically autonomic neuropathy, peripheral neuropathy, CKD, diabetic foot ulcers and retinopathy. There is generally a paucity of large‐scale studies assessing the impact of OSA on diabetes‐related microvascular complications, and this study represents the largest study trying to address this. Previously, Zhang et al. demonstrated the degree of nocturnal hypoxia in individuals with OSA and T2D directly impairs renal function with estimated glomerular filtration rate (eGFR) independently correlating with oxygen desaturation index but failed to demonstrate a significant relationship between severity of OSA and another microvascular complication, retinopathy.^21^ This finding was further confirmed in a cohort study by Tahrani et al., who demonstrated development of OSA in individuals with T2D was independently associated with both the development of diabetic nephropathy and a faster rate of decline in renal function over an average follow up period of 2.5 years.^22^ With respect to peripheral and autonomic neuropathy, the independent association of OSA as a contributing factor has been evidenced previously,^23^ although data quality is generally poor. A systematic review by Abelleira et al. highlighted a lack of longitudinal data, heterogenous design and small sample sizes as limitations in the ability of researchers to draw significant conclusions; however, they conclude a relationship exists between OSA with T2D and occurrence of peripheral neuropathy.^24^ The literature on the impact of OSA on retinopathy development in individuals with T2D is mixed, with several studies demonstrating no significant relationship.^21^, ^25^, ^26^ Conversely, an extensive systematic review and meta‐analysis by Simonson et al. identified a strong association between OSA and retinopathy, but due to the cross‐sectional natures of most of the studies, they were unable to comment on causality.^27^

A novel inclusion in this study was assessing the impact of OSA on the development of MASLD (previously termed non‐alcoholic fatty liver disease) and dementia in individuals with T2D. OSA is a known risk factor for the development of MASLD^11^, ^28^ and dementia^29^ with previous studies focusing on exploring its effect in the general population. We believe this is the largest study demonstrating this same effect in a population of individuals with T2D. We also demonstrate an increased association in rates of MASH in individuals with T2D who develop OSA. Conversely, it is an expected finding individuals with OSA who develop T2D have an increased risk of MASLD, MASH and dementia^30^ as T2D has been a well‐established, independent risk factor for these conditions.^31^, ^32^

Finally, we also looked at the impact a codiagnosis of OSA and T2D on the risk of obesity‐related cancers.^33^ The greatest significant increase in risk was seen for both pancreatic and liver cancer in individuals with OSA who developed T2D with relatively small, but still significant, increases in risk of renal, colon and endometrial cancers. These results should be interpreted within the constraints of this style of analysis and should be seen as identifying significant association rather than causation. However, the findings from this study largely mirror the findings from previous research examining the impact of diabetes on cancer risk.^34^ The impact of a diagnosis of OSA in individuals with T2D was more trivial, but significant associations were still seen for renal and breast cancer. The general relationship between OSA and cancer risk is still relatively unclear in the literature; although largely accepted to increase overall cancer risk of,^35^, ^36^ the effect on specific cancer types is less clear.

The strengths of this study are the size of the population being assessed and the comprehensive array of outcomes explored over a moderate time frame. The limitations reflect the nature of using real‐world data, with data neither randomized nor controlled. Specifically, a significant limitation is that a proportion of patients with T2D and/or OSA are currently undiagnosed. This potentially leads to patients being included in the incorrect cohort and is a limitation inherent to retrospective real‐world studies. Additionally, clinical practices at the HCOs may differ when it comes to investigating, diagnosing and coding the different conditions and outcomes in this study. As the study only work with aggregated results, it is not possible to account for variation in practice between HCOs. Separately, we were unable to account for the effect of smoking or social economic status when designing this study, as this information is poorly captured in the TriNetX network. Additionally, we are reliant on electronic medical records, so data may be incomplete or absent. Data related to mortality are collected only from the electronic medical records and not from government sources and as such may be incomplete. With regard to microvascular outcomes, there is no record of how these diagnoses were made or the criteria/tests used, as such we are unable to account for variation in clinician habits when examining and reporting these health outcomes. Specifically, as microvascular complications are not routinely screened for in an OSA population without concomitant T2D, there may be under reporting of these conditions in that cohort. Other limitations relate to the inability to account for OSA severity, absent BMI data at baseline, CPAP initiation or variation in prescribing habits in individuals with T2D after the index event. We have performed several sensitivity analyses excluding patients who commenced CPAP during the study period, commenced SGLT2i or GLP‐1RA, and one that only included patients with BMI data at baseline, there was no significant difference in the results for cardiovascular, microvascular, liver, all‐cause mortality or dementia outcomes (Tables [Link], [Link]). There are small changes in the cancer outcomes, with supplemental analyses that had a reduced cohort size having wider confidence intervals due to fewer reported outcomes (Tables [Link], [Link], [Link], [Link]), and those with a longer time frame having smaller confidence intervals due to an increased number of outcomes (Tables S2 and S3), no result changed direction.

In conclusion, individuals who codevelop OSA and T2D have a significant increased risk for all CVE, dementia and MASLD, above that seen with either condition in isolation. OSA, in individuals with T2D, increases the risk for autonomic and peripheral neuropathy, diabetic retinopathy and diabetic nephropathy than in individuals with T2D, but no OSA. Both conditions have a significant association with certain obesity‐related malignancies. With the growing armament of licensed and pipeline antiobesity medication, the cumulative risk of these two common obesity‐related comorbidities is relevant in accurately risk stratifying patients and targeting highest risk individuals accordingly. The findings in this study will need confirmation in prospective longitudinal studies.

## AUTHOR CONTRIBUTIONS

DRR contributed to the generation of the results and analysis using the TriNetX platform and took lead on writing the manuscript. AH assisted with the writing, reviewing and editing of the manuscript. MA assisted with reviewing and editing of the manuscript. GH facilitated access to the TriNetX platform and assisted in generating the results, analysis and review of the final manuscript. SSZ provided statistical support and review of the manuscript. UA, SC and JPHW provided senior author review of the manuscript, and DJC oversaw the study development, manuscript writing and provided senior author review of the manuscript. DJC is also the guarantor of this work and had full access to all the data and takes responsibility for the integrity of the data and the accuracy of the data analysis.

## FUNDING INFORMATION

No funding was received for this work.

## CONFLICT OF INTEREST STATEMENT

DRR reports no conflict of interest. GH is an employee of TriNetX LLC. SSZ reports no conflict of interest. AH reports no conflict of interest. MA reports no conflict of interest. SC reports no conflict of interest. DJC has received investigator‐initiated grants from Astra Zeneca and Novo Nordisk and support for education from Perspectum. JPHW reports consultancy/advisory board work for the pharmaceutical industry contracted via the University of Liverpool (no personal payment) for Altimmune, AstraZeneca, Boehringer Ingelheim, Cytoki, Lilly, Napp, Novo Nordisk, Menarini, Pfizer, Rhythm Pharmaceuticals, Sanofi, Saniona, Tern and Shionogi; research grants for clinical trials from AstraZeneca and Novo Nordisk and personal honoraria/lecture fees from AstraZeneca, Boehringer Ingelheim, Medscape, Napp, Novo Nordisk and Rhythm. He is past president of the World Obesity Federation, a member of the Association for the Study of Obesity, Diabetes UK, EASD, ADA, Society for Endocrinology and the Rank Prize Funds Nutrition Committee. From 2009 to 2024, he was national lead for the Metabolic and Endocrine Speciality Group of the UK NIHR Clinical Research Network. UA has received honoraria from Procter & Gamble, Viatris, Grunenthal, Eli Lilly and Sanofi for educational meetings. UA has also received investigator‐led funding by Procter & Gamble.

### PEER REVIEW

The peer review history for this article is available at https://www.webofscience.com/api/gateway/wos/peer-review/10.1111/dom.16059.

## ETHICS STATEMENT

No ethical approval is required for this study. As a federated network, research studies using TriNetX do not require ethical approval. To comply with legal frameworks and ethical guidelines guarding against data reidentification, the identity of participating healthcare organizations and their individual contribution to each dataset are not disclosed. The TriNetX platform only uses aggregated counts and statistical summaries of deidentified information. No protected health information or personal data are made available to the users of the platform.

## Supporting information


**Supplemental Table S1.** Codes used for outcome analyses.

**Supplemental Table S2.** Sensitivity analysis of analysis one (OSA+T2D vs. OSA)—This analysis uses the same methodology as described in the main manuscript with the only addition that the time window is from 1 to 1825 days.

**Supplemental Table S3.** Sensitivity analysis of analysis two (T2D+OSA vs. T2D)—This analysis uses the same methodology as described in the main manuscript with the only addition that the time window is from 1 to 1825 days.

**Supplemental Table S4.** Sensitivity analysis of analysis one (OSA+T2D vs. OSA)—This analysis uses the same methodology as described in the main manuscript with the only addition that patients are required to have BMI data available at baseline.

**Supplemental Table S5.** Sensitivity analysis of analysis two (T2D+OSA vs. T2D)—This analysis uses the same methodology as described in the main manuscript with the only addition that patients are required to have both BMI and HbA1c data available at baseline.

**Supplemental Table S6.** Sensitivity analysis of analysis one (OSA+T2D vs. OSA)—This analysis uses the same methodology as described in the main manuscript with the only change that patients who initiated CPAP are excluded.

**Supplemental Table S7.** Sensitivity analysis of analysis two (T2D+OSA vs. T2D)—This analysis uses the same methodology as described in the main manuscript with the only change is that patients prescribed SGLT2i or GLP‐1RA prior to or within the time window are excluded.

## Data Availability

The data that support the findings of this study are available from TriNetX, LLC, https://trinetx.com/, but third‐party restrictions apply to the availability of these data. The data were used under licence for this study with restrictions that do not allow for the data to be redistributed or made publicly available. However, for accredited researchers, the TriNetX data are available for licensing at TriNetX, LLC. Data access may require a data sharing agreement and may incur data access fees. Data used in the generation of this paper was collected from the global TriNetX network, and local data at LUHFT were not used.
