# Supplementary material for: The cumulative impact of type 2 diabetes and obstructive sleep apnoea on cardiovascular, liver, diabetes‐related and cancer outcomes
Source: Diabetes Obes Metab. 2024 Nov 11;27(2):663–74. doi: 10.1111/dom.16059 (PMC11701193; doi:10.1111/dom.16059)
Supplement: Supplementary file 1 — Supplemental Table S1. Codes used for outcome analyses. [file DOM-27-663-s007.docx]

**Supplemental table 1 – Codes used for outcome analyses.**

| Peripheral Neuropathy | | | | |
| --- | --- | --- | --- | --- |
|  | **Outcome definition** | | | |
|  | | Diagnosis | UMLS:ICD10CM:E11.42 | Type 2 diabetes mellitus with diabetic polyneuropathy |
|  | | Diagnosis | UMLS:ICD10CM:E11.40 | Type 2 diabetes mellitus with diabetic neuropathy, unspecified |
|  | | Diagnosis | UMLS:ICD10CM:E11.41 | Type 2 diabetes mellitus with diabetic mononeuropathy |
|  | | Diagnosis | UMLS:ICD10CM:G62.9 | Polyneuropathy, unspecified |
|  | | Diagnosis | UMLS:ICD10CM:G59 | Mononeuropathy in diseases classified elsewhere |
|  | **Settings for the performed analyses** | | | |
|  | | Kaplan - Meier survival analysis | | excluding patients with outcome prior to the time window |
| Macular Oedema | | | | |
|  | **Outcome definition** | | | |
|  | | Diagnosis | UMLS:ICD10CM:H35.81 | Retinal edema |
|  | | Diagnosis | UMLS:ICD10CM:E11.311 | Type 2 diabetes mellitus with unspecified diabetic retinopathy with macular edema |
|  | | Diagnosis | UMLS:ICD10CM:E11.321 | Type 2 diabetes mellitus with mild nonproliferative diabetic retinopathy with macular edema |
|  | | Diagnosis | UMLS:ICD10CM:E11.331 | Type 2 diabetes mellitus with moderate nonproliferative diabetic retinopathy with macular edema |
|  | | Diagnosis | UMLS:ICD10CM:E11.341 | Type 2 diabetes mellitus with severe nonproliferative diabetic retinopathy with macular edema |
|  | | Diagnosis | UMLS:ICD10CM:E11.351 | Type 2 diabetes mellitus with proliferative diabetic retinopathy with macular edema |
|  | **Settings for the performed analyses** | | | |
|  | | Kaplan - Meier survival analysis | | excluding patients with outcome prior to the time window |
| Retinopathy with no Macular Oedema | | | | |
|  | **Outcome definition** | | | |
|  | | Diagnosis | UMLS:ICD10CM:E11.319 | Type 2 diabetes mellitus with unspecified diabetic retinopathy without macular edema |
|  | | Diagnosis | UMLS:ICD10CM:E11.329 | Type 2 diabetes mellitus with mild nonproliferative diabetic retinopathy without macular edema |
|  | | Diagnosis | UMLS:ICD10CM:E11.352 | Type 2 diabetes mellitus with proliferative diabetic retinopathy with traction retinal detachment involving the macula |
|  | | Diagnosis | UMLS:ICD10CM:E11.353 | Type 2 diabetes mellitus with proliferative diabetic retinopathy with traction retinal detachment not involving the macula |
|  | | Diagnosis | UMLS:ICD10CM:E11.354 | Type 2 diabetes mellitus with proliferative diabetic retinopathy with combined traction retinal detachment and rhegmatogenous retinal detachment |
|  | | Diagnosis | UMLS:ICD10CM:E11.355 | Type 2 diabetes mellitus with stable proliferative diabetic retinopathy |
|  | | Diagnosis | UMLS:ICD10CM:E11.359 | Type 2 diabetes mellitus with proliferative diabetic retinopathy without macular edema |
|  | | Diagnosis | UMLS:ICD10CM:E11.339 | Type 2 diabetes mellitus with moderate nonproliferative diabetic retinopathy without macular edema |
|  | | Diagnosis | UMLS:ICD10CM:E11.349 | Type 2 diabetes mellitus with severe nonproliferative diabetic retinopathy without macular edema |
|  | | Diagnosis | UMLS:ICD10CM:H35.00 | Unspecified background retinopathy |
|  | | Diagnosis | UMLS:ICD10CM:H35.01 | Changes in retinal vascular appearance |
|  | | Diagnosis | UMLS:ICD10CM:H35.02 | Exudative retinopathy |
|  | | Diagnosis | UMLS:ICD10CM:H35.03 | Hypertensive retinopathy |
|  | | Diagnosis | UMLS:ICD10CM:H35.04 | Retinal micro-aneurysms, unspecified |
|  | | Diagnosis | UMLS:ICD10CM:H35.05 | Retinal neovascularization, unspecified |
|  | | Diagnosis | UMLS:ICD10CM:H35.09 | Other intraretinal microvascular abnormalities |
|  | **Settings for the performed analyses** | | | |
|  | | Kaplan - Meier survival analysis | | excluding patients with outcome prior to the time window |
| Amputations | | | | |
|  | **Outcome definition** | | | |
|  | | Procedure | UMLS:CPT:1014590 | Amputation, leg, through tibia and fibula |
|  | | Procedure | UMLS:CPT:1005524 | Amputation Procedures on the Foot and Toes |
|  | | Procedure | UMLS:CPT:28820 | Amputation, toe; metatarsophalangeal joint |
|  | | Procedure | UMLS:CPT:1005298 | Amputation Procedures on the Leg (Tibia and Fibula) and Ankle Joint |
|  | | Procedure | UMLS:CPT:1005529 | Amputation, toe |
|  | | Procedure | UMLS:SNOMED:397117006 | Amputation of lower limb |
|  | | Procedure | UMLS:CPT:28810 | Amputation, metatarsal, with toe, single |
|  | | Procedure | UMLS:CPT:27880 | Amputation, leg, through tibia and fibula |
|  | | Procedure | UMLS:CPT:1005146 | Amputation Procedures on the Femur (Thigh Region) and Knee Joint |
|  | | Procedure | UMLS:CPT:1014580 | Amputation, thigh, through femur, any level |
|  | | Procedure | UMLS:CPT:27590 | Amputation, thigh, through femur, any level |
|  | | Procedure | UMLS:SNOMED:180030006 | Amputation of the foot |
|  | | Procedure | UMLS:CPT:1005525 | Amputation, foot |
|  | | Procedure | UMLS:SNOMED:371186005 | Amputation of toe |
|  | | Procedure | UMLS:CPT:28805 | Amputation, foot; transmetatarsal |
|  | | Procedure | UMLS:SNOMED:400053006 | Amputation of limb |
|  | | Procedure | UMLS:CPT:27882 | Amputation, leg, through tibia and fibula; open, circular (guillotine) |
|  | | Procedure | UMLS:CPT:27886 | Amputation, leg, through tibia and fibula; re-amputation |
|  | | Procedure | UMLS:SNOMED:88312006 | Amputation of leg through tibia and fibula |
|  | | Procedure | UMLS:CPT:27596 | Amputation, thigh, through femur, any level; re-amputation |
|  | | Procedure | UMLS:CPT:27884 | Amputation, leg, through tibia and fibula; secondary closure or scar revision |
|  | | Procedure | UMLS:SNOMED:180038004 | Amputation of phalanx of toe |
|  | | Procedure | UMLS:SNOMED:265739006 | Amputation through metatarsal bones |
|  | | Procedure | UMLS:CPT:27592 | Amputation, thigh, through femur, any level; open, circular (guillotine) |
|  | | Procedure | UMLS:CPT:28800 | Amputation, foot; midtarsal (eg, Chopart type procedure) |
|  | | Procedure | UMLS:CPT:27881 | Amputation, leg, through tibia and fibula; with immediate fitting technique including application of first cast |
|  | | Procedure | UMLS:SNOMED:110470001 | Amputation above-knee, mid-thigh |
|  | | Procedure | UMLS:CPT:27591 | Amputation, thigh, through femur, any level; immediate fitting technique including first cast |
|  | **Settings for the performed analyses** | | | |
|  | | Kaplan - Meier survival analysis | | excluding patients with outcome prior to the time window |
| Autonomic Neuropathy | | | | |
|  | **Outcome definition** | | | |
|  | | Diagnosis | UMLS:ICD10CM:E11.43 | Type 2 diabetes mellitus with diabetic autonomic (poly)neuropathy |
|  | | Diagnosis | UMLS:ICD10CM:G99.0 | Autonomic neuropathy in diseases classified elsewhere |
|  | | Diagnosis | UMLS:ICD10CM:E13.43 | Other specified diabetes mellitus with diabetic autonomic (poly)neuropathy |
|  | **Settings for the performed analyses** | | | |
|  | | Kaplan - Meier survival analysis | | excluding patients with outcome prior to the time window |
| CKD | | | | |
|  | **Outcome definition** | | | |
|  | | Diagnosis | UMLS:ICD10CM:N18 | Chronic kidney disease (CKD) |
|  | | Diagnosis | UMLS:ICD10CM:E11.22 | Type 2 diabetes mellitus with diabetic chronic kidney disease |
|  | **Settings for the performed analyses** | | | |
|  | | Kaplan - Meier survival analysis | | excluding patients with outcome prior to the time window |
| All-cause mortality | | | | |
|  | **Outcome definition** | | | |
|  | | Demographics | Deceased | Deceased |
|  | | Diagnosis | UMLS:ICD10CM:R99 | Ill-defined and unknown cause of mortality |
|  | **Settings for the performed analyses** | | | |
|  | | Kaplan - Meier survival analysis | | including patients with outcome prior to the time window |
| IHD | | | | |
|  | **Outcome definition** | | | |
|  | | Diagnosis | UMLS:ICD10CM:I20-I25 | Ischemic heart diseases |
|  | **Settings for the performed analyses** | | | |
|  | | Kaplan - Meier survival analysis | | excluding patients with outcome prior to the time window |
| Heart Failure | | | | |
|  | **Outcome definition** | | | |
|  | | Diagnosis | UMLS:ICD10CM:I50 | Heart failure |
|  | **Settings for the performed analyses** | | | |
|  | | Kaplan - Meier survival analysis | | excluding patients with outcome prior to the time window |
| Dementia | | | | |
|  | **Outcome definition** | | | |
|  | | Diagnosis | UMLS:ICD10CM:F03 | Unspecified dementia |
|  | | Diagnosis | UMLS:ICD10CM:F01 | Vascular dementia |
|  | | Diagnosis | UMLS:ICD10CM:F02 | Dementia in other diseases classified elsewhere |
|  | | Diagnosis | UMLS:ICD10CM:G30 | Alzheimer's disease |
|  | **Settings for the performed analyses** | | | |
|  | | Kaplan - Meier survival analysis | | excluding patients with outcome prior to the time window |
| Atrial fibrillation and flutter | | | | |
|  | **Outcome definition** | | | |
|  | | Diagnosis | UMLS:ICD10CM:I48 | Atrial fibrillation and flutter |
|  | **Settings for the performed analyses** | | | |
|  | | Kaplan - Meier survival analysis | | excluding patients with outcome prior to the time window |
| Ischaemic stroke | | | | |
|  | **Outcome definition** | | | |
|  | | Diagnosis | UMLS:ICD10CM:I63 | Cerebral infarction |
|  | **Settings for the performed analyses** | | | |
|  | | Kaplan - Meier survival analysis | | excluding patients with outcome prior to the time window |
| NAFLD | | | | |
|  | **Outcome definition** | | | |
|  | | Diagnosis | UMLS:ICD10CM:K76.0 | Fatty (change of) liver, not elsewhere classified |
|  | **Settings for the performed analyses** | | | |
|  | | Kaplan - Meier survival analysis | | excluding patients with outcome prior to the time window |
| NASH | | | | |
|  | **Outcome definition** | | | |
|  | | Diagnosis | UMLS:ICD10CM:K75.81 | Nonalcoholic steatohepatitis (NASH) |
|  | **Settings for the performed analyses** | | | |
|  | | Kaplan - Meier survival analysis | | excluding patients with outcome prior to the time window |
| liver cancer | | | | |
|  | **Outcome definition** | | | |
|  | | Diagnosis | UMLS:ICD10CM:C22.0 | Liver cell carcinoma |
|  | | Diagnosis | UMLS:ICD10CM:C22.8 | Malignant neoplasm of liver, primary, unspecified as to type |
|  | **Settings for the performed analyses** | | | |
|  | | Kaplan - Meier survival analysis | | excluding patients with outcome prior to the time window |
| Pancreatic cancer | | | | |
|  | **Outcome definition** | | | |
|  | | Diagnosis | UMLS:ICD10CM:C25 | Malignant neoplasm of pancreas |
|  | **Settings for the performed analyses** | | | |
|  | | Kaplan - Meier survival analysis | | excluding patients with outcome prior to the time window |
| breast cancer | | | | |
|  | **Outcome definition** | | | |
|  | | Diagnosis | UMLS:ICD10CM:C50 | Malignant neoplasm of breast |
|  | **Settings for the performed analyses** | | | |
|  | | Kaplan - Meier survival analysis | | excluding patients with outcome prior to the time window |
| Colon cancer | | | | |
|  | **Outcome definition** | | | |
|  | | Diagnosis | UMLS:ICD10CM:C18 | Malignant neoplasm of colon |
|  | **Settings for the performed analyses** | | | |
|  | | Kaplan - Meier survival analysis | | excluding patients with outcome prior to the time window |
| Diabetic Ulcer | | | | |
|  | **Outcome definition** | | | |
|  | | Diagnosis | UMLS:ICD10CM:E11.621 | Type 2 diabetes mellitus with foot ulcer |
|  | | Diagnosis | UMLS:ICD10CM:L97.4 | Non-pressure chronic ulcer of heel and midfoot |
|  | | Diagnosis | UMLS:ICD10CM:L97.5 | Non-pressure chronic ulcer of other part of foot |
|  | **Settings for the performed analyses** | | | |
|  | | Kaplan - Meier survival analysis | | excluding patients with outcome prior to the time window |
| Gallbladder cancer | | | | |
|  | **Outcome definition** | | | |
|  | | Diagnosis | UMLS:ICD10CM:C23 | Malignant neoplasm of gallbladder |
|  | **Settings for the performed analyses** | | | |
|  | | Kaplan - Meier survival analysis | | excluding patients with outcome prior to the time window |
| Renal cancer | | | | |
|  | **Outcome definition** | | | |
|  | | Diagnosis | UMLS:ICD10CM:C65 | Malignant neoplasm of renal pelvis |
|  | | Diagnosis | UMLS:ICD10CM:C64 | Malignant neoplasm of kidney, except renal pelvis |
|  | **Settings for the performed analyses** | | | |
|  | | Kaplan - Meier survival analysis | | excluding patients with outcome prior to the time window |
| Oesophageal cancer | | | | |
|  | **Outcome definition** | | | |
|  | | Diagnosis | UMLS:ICD10CM:C15 | Malignant neoplasm of esophagus |
|  | **Settings for the performed analyses** | | | |
|  | | Kaplan - Meier survival analysis | | excluding patients with outcome prior to the time window |
| Endometrial cancer | | | | |
|  | **Outcome definition** | | | |
|  | | Diagnosis | UMLS:ICD10CM:C54 | Malignant neoplasm of corpus uteri |
|  | **Settings for the performed analyses** | | | |
|  | | Kaplan - Meier survival analysis | | excluding patients with outcome prior to the time window |
