# Supplementary material for: The cumulative impact of type 2 diabetes and obstructive sleep apnoea on cardiovascular, liver, diabetes‐related and cancer outcomes
Source: Diabetes Obes Metab. 2024 Nov 11;27(2):663–74. doi: 10.1111/dom.16059 (PMC11701193; doi:10.1111/dom.16059)
Supplement: Supplementary file 2 — Supplemental Table S2. Sensitivity analysis of analysis one (OSA+T2D vs. OSA)—This analysis uses the same methodology as described in the main manuscript with the only addition that the time window is from 1 to 1825 days. [file DOM-27-663-s003.docx]

**Supplemental Table 2 – Sensitivity analysis of analysis one (OSA+T2D vs OSA) –** This analysis uses the same methodology as described in the main manuscript with the only addition that the time window is from 1 to 1825 days.

|  | **Cohorts** | **Sample size** ^†^ | **Outcome** | **Hazard ratio** | **95% CI** |
| --- | --- | --- | --- | --- | --- |
| Peripheral neuropathy | OSA + T2D | 167,079 | 19,283 | 3.676 | (3.573, 3.783) |
|  | OSA | 175,707 | 6,232 |  |  |
| Macular oedema | OSA + T2D | 179,059 | 1,672 | 8.425 | (7.310, 9.711) |
|  | OSA | 179,482 | 215 |  |  |
| Retinopathy (excluding macular oedema) | OSA + T2D | 177,566 | 4,608 | 6.688 | (6.194, 7.222) |
|  | OSA | 179,010 | 758 |  |  |
| Amputations | OSA + T2D | 179,579 | 667 | 4.684 | (3.927, 5.586) |
|  | OSA | 179,604 | 152 |  |  |
| Autonomic neuropathy | OSA + T2D | 179,069 | 1,769 | 47.032 | (34.509, 64.101) |
|  | OSA | 179,659 | 41 |  |  |
| CKD | OSA + T2D | 152,297 | 19,333 | 2.192 | (2.139, 2.245) |
|  | OSA | 155,217 | 9,923 |  |  |
| Foot ulcers | OSA + T2D | 177,374 | 5,302 | 6.540 | (6.092, 7.020) |
|  | OSA | 179,140 | 894 |  |  |
| **Cardiovascular outcomes** | **Cohorts** | **Sample size** ^†^ | **Outcome** | **Hazard ratio** | **95% CI** |
| Ischaemic heart disease | OSA + T2D | 130,043 | 22,269 | 1.825 | (1.786, 1.864) |
|  | OSA | 131,447 | 13,666 |  |  |
| Heart failure | OSA + T2D | 144,189 | 19,249 | 1.827 | (1.787, 1.869) |
|  | OSA | 155,887 | 12,432 |  |  |
| Atrial fibrillation | OSA + T2D | 150,236 | 13,303 | 1.480 | (1.442, 1.519) |
|  | OSA | 152,383 | 9,846 |  |  |
| Ischaemic stroke | OSA + T2D | 172,972 | 6,385 | 1.566 | (1.507, 1.627) |
|  | OSA | 173,838 | 4,435 |  |  |
| **Neoplastic outcomes** | **Cohorts** | **Sample size** ^†^ | **Outcome** | **Hazard ratio** | **95% CI** |
| Liver cancer | OSA + T2D | 179,319 | 441 | 2.235 | (1.898, 2.633) |
|  | OSA | 179,479 | 212 |  |  |
| Pancreatic cancer | OSA + T2D | 179,094 | 588 | 2.037 | (1.776, 2.338) |
|  | OSA | 179,445 | 310 |  |  |
| Breast cancer | OSA + T2D | 177,843 | 1,430 | 1.159 | (1.076, 1.249) |
|  | OSA | 177,143 | 1,326 |  |  |
| Colon cancer | OSA + T2D | 178,804 | 878 | 1.471 | (1.328, 1.628) |
|  | OSA | 178,691 | 643 |  |  |
| Cholangiocarcinoma | OSA + T2D | 179,668 | 43 | 1.396 | (0.887, 2.198) |
|  | OSA | 179,658 | 33 |  |  |
| Renal cancer | OSA + T2D | 178,626 | 939 | 1.299 | (1.181, 1.428) |
|  | OSA | 178,458 | 776 |  |  |
| Oesophageal cancer | OSA + T2D | 179,410 | 215 | 1.338 | (1.095, 1.634) |
|  | OSA | 179,449 | 173 |  |  |
| Endometrial cancer | OSA + T2D | 178,964 | 491 | 1.440 | (1.257, 1.648) |
|  | OSA | 179,025 | 367 |  |  |
| **All-cause mortality, dementia and liver outcomes** | **Cohorts** | **Sample size** ^†^ | **Outcome** | **Hazard ratio** | **95% CI** |
| All-cause mortality | OSA + T2D | 179,688 | 24,002 | 1.535 | (1.505, 1.565) |
|  | OSA | 179,688 | 16,890 |  |  |
| Dementia | OSA + T2D | 176,780 | 3,953 | 1.286 | (1.228, 1.347) |
|  | OSA | 176,896 | 3,327 |  |  |
| Metabolic dysfunction-associated steatotic liver disease | OSA + T2D | 173,490 | 10,911 | 1.858 | (1.802, 1.916) |
|  | OSA | 174,783 | 6,518 |  |  |
| Metabolic dysfunction-associated steatohepatitis | OSA + T2D | 178,424 | 2,388 | 2.848 | (2.639, 3.073) |
|  | OSA | 179,158 | 913 |  |  |

T2D: Type 2 diabetes. OSA: Obstructive sleep apnoea. CKD: Chronic kidney disease. ^†^number of participants
