# Supplementary material for: The cumulative impact of type 2 diabetes and obstructive sleep apnoea on cardiovascular, liver, diabetes‐related and cancer outcomes
Source: Diabetes Obes Metab. 2024 Nov 11;27(2):663–74. doi: 10.1111/dom.16059 (PMC11701193; doi:10.1111/dom.16059)
Supplement: Supplementary file 3 — Supplemental Table S3. Sensitivity analysis of analysis two (T2D+OSA vs. T2D)—This analysis uses the same methodology as described in the main manuscript with the only addition that the time window is from 1 to 1825 days. [file DOM-27-663-s002.docx]

**Supplemental Table 3 – Sensitivity analysis of analysis two (T2D+OSA vs T2D) –** This analysis uses the same methodology as described in the main manuscript with the only addition that the time window is from 1 to 1825 days.

|  | **Cohorts** | **Sample size** ^†^ | **Outcome** | **Hazard ratio** | **95% CI** |
| --- | --- | --- | --- | --- | --- |
| Peripheral neuropathy | T2D + OSA | 221,877 | 32,904 | 1.621 | (1.594, 1.650) |
|  | T2D | 227,639 | 21,353 |  |  |
| Macular oedema | T2D + OSA | 238,897 | 3,578 | 1.081 | (1.031, 1.133) |
|  | T2D | 238,306 | 3,264 |  |  |
| Retinopathy (excluding macular oedema) | T2D + OSA | 236,646 | 9,531 | 1.265 | (1.228, 1.304) |
|  | T2D | 236,105 | 7,471 |  |  |
| Amputations | T2D + OSA | 239,954 | 1,204 | 1.019 | (0.940, 1.105) |
|  | T2D | 239,865 | 1,166 |  |  |
| Autonomic neuropathy | T2D + OSA | 239,158 | 3,308 | 1.800 | (1.700, 1.906) |
|  | T2D | 239,468 | 1,828 |  |  |
| CKD | T2D + OSA | 204,756 | 32,814 | 1.484 | (1.459, 1.510) |
|  | T2D | 204,817 | 22,543 |  |  |
| Foot ulcers | T2D + OSA | 236,326 | 9,097 | 1.292 | (1.252, 1.333) |
|  | T2D | 236,188 | 7,002 |  |  |
| **Cardiovascular outcomes** | **Cohorts** | **Sample size** ^†^ | **Outcome** | **Hazard ratio** | **95% CI** |
| Ischaemic heart disease | T2D + OSA | 178,173 | 37,970 | 1.545 | (1.521, 1.570) |
|  | T2D | 178,245 | 25,382 |  |  |
| Heart failure | T2D + OSA | 197,459 | 32,999 | 1.682 | (1.653, 1.711) |
|  | T2D | 212,511 | 21,508 |  |  |
| Atrial fibrillation | T2D + OSA | 204,934 | 22,255 | 1.529 | (1.498, 1.561) |
|  | T2D | 216,890 | 15,450 |  |  |
| Ischaemic stroke | T2D + OSA | 231,358 | 10,388 | 1.302 | (1.264, 1.340) |
|  | T2D | 232,104 | 7,982 |  |  |
| **Neoplastic outcomes** | **Cohorts** | **Sample size** ^†^ | **Outcome** | **Hazard ratio** | **95% CI** |
| Liver cancer | T2D + OSA | 239,573 | 720 | 1.073 | (0.966, 1.193) |
|  | T2D | 239,459 | 663 |  |  |
| Pancreatic cancer | T2D + OSA | 239,364 | 884 | 1.138 | (1.033, 1.254) |
|  | T2D | 239,354 | 767 |  |  |
| Breast cancer | T2D + OSA | 237,693 | 2,310 | 1.263 | (1.187, 1.343) |
|  | T2D | 237,545 | 1,811 |  |  |
| Colon cancer | T2D + OSA | 238,910 | 1,382 | 1.115 | (1.033, 1.205) |
|  | T2D | 238,815 | 1,225 |  |  |
| Cholangiocarcinoma | T2D + OSA | 240,064 | 60 | 0.883 | (0.623, 1.251) |
|  | T2D | 240,059 | 67 |  |  |
| Renal cancer | T2D + OSA | 238,737 | 1,577 | 1.353 | (1.254, 1.459) |
|  | T2D | 238,988 | 1,153 |  |  |
| Oesophageal cancer | T2D + OSA | 239,700 | 342 | 1.262 | (1.076, 1.481) |
|  | T2D | 239,776 | 268 |  |  |
| Endometrial cancer | T2D + OSA | 239,124 | 786 | 1.208 | (1.089, 1.341) |
|  | T2D | 239,120 | 643 |  |  |
| **All-cause mortality, dementia and liver outcomes** | **Cohorts** | **Sample size** ^†^ | **Outcome** | **Hazard ratio** | **95% CI** |
| All-cause mortality | T2D + OSA | 240,094 | 33,792 | 1.181 | (1.163, 1.200) |
|  | T2D | 240,094 | 28,454 |  |  |
| Dementia | T2D + OSA | 236,643 | 6,543 | 1.309 | (1.261, 1.358) |
|  | T2D | 236,531 | 4,973 |  |  |
| Metabolic dysfunction-associated steatotic liver disease | T2D + OSA | 232,794 | 16,443 | 1.614 | (1.575, 1.654) |
|  | T2D | 234,098 | 10,325 |  |  |
| Metabolic dysfunction-associated steatohepatitis | T2D + OSA | 238,518 | 3,743 | 1.781 | (1.688, 1.879) |
|  | T2D | 239,054 | 2,093 |  |  |

T2D: Type 2 diabetes. OSA: Obstructive sleep apnoea. CKD: Chronic kidney disease. ^†^number of participants
