# Supplementary material for: The cumulative impact of type 2 diabetes and obstructive sleep apnoea on cardiovascular, liver, diabetes‐related and cancer outcomes
Source: Diabetes Obes Metab. 2024 Nov 11;27(2):663–74. doi: 10.1111/dom.16059 (PMC11701193; doi:10.1111/dom.16059)
Supplement: Supplementary file 4 — Supplemental Table S4. Sensitivity analysis of analysis one (OSA+T2D vs. OSA)—This analysis uses the same methodology as described in the main manuscript with the only addition that patients are required to have BMI data available at baseline. [file DOM-27-663-s004.docx]

**Supplemental Table 4 – Sensitivity analysis of analysis one (OSA+T2D vs OSA) –** This analysis uses the same methodology as described in the main manuscript with the only addition that patients are required to have BMI data available at baseline.

|  | **Cohorts** | **Sample size** ^†^ | **Outcome** | **Hazard ratio** | **95% CI** |
| --- | --- | --- | --- | --- | --- |
| Peripheral neuropathy | OSA + T2D | 134,759 | 8,480 | 3.004 | (2.887, 3.125) |
|  | OSA | 145,881 | 3,468 |  |  |
| Macular oedema | OSA + T2D | 149,250 | 746 | 6.636 | (5.494, 8.015) |
|  | OSA | 150,197 | 126 |  |  |
| Retinopathy (excluding macular oedema) | OSA + T2D | 147,195 | 2,153 | 6.063 | (5.452, 6.743) |
|  | OSA | 149,676 | 404 |  |  |
| Amputations | OSA + T2D | 150,096 | 205 | 3.745 | (2.814, 4.984) |
|  | OSA | 150,294 | 61 |  |  |
| Autonomic neuropathy | OSA + T2D | 149,465 | 786 | 35.225 | (23.655, 52.455) |
|  | OSA | 150,363 | 25 |  |  |
| CKD | OSA + T2D | 123,391 | 7,755 | 1.756 | (1.696, 1.820) |
|  | OSA | 130,002 | 5,145 |  |  |
| Foot ulcers | OSA + T2D | 147,049 | 2,353 | 5.839 | (5.283, 6.453) |
|  | OSA | 149,843 | 459 |  |  |
| **Cardiovascular outcomes** | **Cohorts** | **Sample size** ^†^ | **Outcome** | **Hazard ratio** | **95% CI** |
| Ischaemic heart disease | OSA + T2D | 102,815 | 8,359 | 1.407 | (1.363, 1.453) |
|  | OSA | 108,557 | 6,905 |  |  |
| Heart failure | OSA + T2D | 118,012 | 7,129 | 1.476 | (1.426, 1.529) |
|  | OSA | 129,777 | 5,806 |  |  |
| Atrial fibrillation | OSA + T2D | 123,337 | 5,046 | 1.151 | (1.107, 1.198) |
|  | OSA | 126,574 | 4,921 |  |  |
| Ischaemic stroke | OSA + T2D | 143,087 | 2,719 | 1.411 | (1.334, 1.493) |
|  | OSA | 144,793 | 2,169 |  |  |
| **Neoplastic outcomes** | **Cohorts** | **Sample size** ^†^ | **Outcome** | **Hazard ratio** | **95% CI** |
| Liver cancer | OSA + T2D | 149,933 | 187 | 2.119 | (1.659, 2.705) |
|  | OSA | 150,181 | 98 |  |  |
| Pancreatic cancer | OSA + T2D | 149,671 | 232 | 1.573 | (1.288, 1.922) |
|  | OSA | 150,132 | 164 |  |  |
| Breast cancer | OSA + T2D | 148,375 | 703 | 0.970 | (0.876, 1.073) |
|  | OSA | 148,003 | 803 |  |  |
| Colon cancer | OSA + T2D | 149,385 | 369 | 1.133 | (0.980, 1.310) |
|  | OSA | 149,434 | 362 |  |  |
| Cholangiocarcinoma | OSA + T2D | 150,371 | 21 | 1.289 | (0.687, 2.419) |
|  | OSA | 150,367 | 18 |  |  |
| Renal cancer | OSA + T2D | 149,220 | 382 | 1.318 | (1.136, 1.528) |
|  | OSA | 149,257 | 322 |  |  |
| Oesophageal cancer | OSA + T2D | 150,089 | 101 | 1.183 | (0.894, 1.565) |
|  | OSA | 150,160 | 95 |  |  |
| Endometrial cancer | OSA + T2D | 149,658 | 218 | 1.188 | (0.982, 1.438) |
|  | OSA | 149,752 | 204 |  |  |
| **All-cause mortality, dementia and liver outcomes** | **Cohorts** | **Sample size** ^†^ | **Outcome** | **Hazard ratio** | **95% CI** |
| All-cause mortality | OSA + T2D | 150,399 | 11,143 | 1.496 | (1.454, 1.539) |
|  | OSA | 150,399 | 8,297 |  |  |
| Dementia | OSA + T2D | 146,975 | 1,815 | 1.171 | (1.097, 1.251) |
|  | OSA | 147,570 | 1,725 |  |  |
| Metabolic dysfunction-associated steatotic liver disease | OSA + T2D | 141,510 | 5,005 | 1.665 | (1.594, 1.738) |
|  | OSA | 144,548 | 3,468 |  |  |
| Metabolic dysfunction-associated steatohepatitis | OSA + T2D | 148,704 | 1,096 | 2.458 | (2.212, 2.733) |
|  | OSA | 149,734 | 500 |  |  |

T2D: Type 2 diabetes. OSA: Obstructive sleep apnoea. CKD: Chronic kidney disease. ^†^number of participants
