# Supplementary material for: The cumulative impact of type 2 diabetes and obstructive sleep apnoea on cardiovascular, liver, diabetes‐related and cancer outcomes
Source: Diabetes Obes Metab. 2024 Nov 11;27(2):663–74. doi: 10.1111/dom.16059 (PMC11701193; doi:10.1111/dom.16059)
Supplement: Supplementary file 5 — Supplemental Table S5. Sensitivity analysis of analysis two (T2D+OSA vs. T2D)—This analysis uses the same methodology as described in the main manuscript with the only addition that patients are required to have both BMI and HbA1c data available at baseline. [file DOM-27-663-s006.docx]

**Supplemental Table 5 – Sensitivity analysis of analysis two (T2D+OSA vs T2D) –** This analysis uses the same methodology as described in the main manuscript with the only addition that patients are required to have both BMI and HbA1c data available at baseline.

|  | **Cohorts** | **Sample size** ^†^ | **Outcome** | **Hazard ratio** | **95% CI** |
| --- | --- | --- | --- | --- | --- |
| Peripheral neuropathy | T2D + OSA | 36,103 | 3,884 | 1.318 | (1.258, 1.382) |
|  | T2D | 37,762 | 3,213 |  |  |
| Macular oedema | T2D + OSA | 41,800 | 442 | 1.035 | (0.907, 1.182) |
|  | T2D | 41,793 | 437 |  |  |
| Retinopathy (excluding macular oedema) | T2D + OSA | 40,922 | 1,222 | 1.149 | (1.059, 1.246) |
|  | T2D | 41,094 | 1,098 |  |  |
| Amputations | T2D + OSA | 42,074 | 143 | 1.202 | (0.944, 1.530) |
|  | T2D | 42,081 | 122 |  |  |
| Autonomic neuropathy | T2D + OSA | 41,830 | 416 | 1.419 | (1.223, 1.645) |
|  | T2D | 41,966 | 302 |  |  |
| CKD | T2D + OSA | 33,155 | 3,369 | 1.190 | (1.133, 1.250) |
|  | T2D | 34,108 | 3,005 |  |  |
| Foot ulcers | T2D + OSA | 40,847 | 1,167 | 1.126 | (1.037, 1.224) |
|  | T2D | 40,949 | 1,066 |  |  |
| **Cardiovascular outcomes** | **Cohorts** | **Sample size** ^†^ | **Outcome** | **Hazard ratio** | **95% CI** |
| Ischaemic heart disease | T2D + OSA | 28,081 | 3,592 | 1.298 | (1.236, 1.362) |
|  | T2D | 29,430 | 3,005 |  |  |
| Heart failure | T2D + OSA | 31,776 | 2,978 | 1.207 | (1.146, 1.271) |
|  | T2D | 34,254 | 2,718 |  |  |
| Atrial fibrillation | T2D + OSA | 34,576 | 2,089 | 1.141 | (1.073, 1.214) |
|  | T2D | 36,336 | 1,961 |  |  |
| Ischaemic stroke | T2D + OSA | 39,334 | 1,117 | 1.141 | (1.048, 1.242) |
|  | T2D | 39,778 | 1,018 |  |  |
| **Neoplastic outcomes** | **Cohorts** | **Sample size** ^†^ | **Outcome** | **Hazard ratio** | **95% CI** |
| Liver cancer | T2D + OSA | 42,066 | 75 | 0.926 | (0.678, 1.265) |
|  | T2D | 42,087 | 83 |  |  |
| Pancreatic cancer | T2D + OSA | 42,051 | 66 | 0.879 | (0.633, 1.221) |
|  | T2D | 42,064 | 77 |  |  |
| Breast cancer | T2D + OSA | 41,568 | 277 | 1.051 | (0.889, 1.243) |
|  | T2D | 41,567 | 270 |  |  |
| Colon cancer | T2D + OSA | 41,936 | 161 | 1.115 | (0.892, 1.394) |
|  | T2D | 41,933 | 148 |  |  |
| Cholangiocarcinoma | T2D + OSA | 42,231 | 10 | 0.848 | (0.259, 2.777) |
|  | T2D | 42,234 | 10 |  |  |
| Renal cancer | T2D + OSA | 41,898 | 166 | 1.058 | (0.852, 1.314) |
|  | T2D | 41,974 | 161 |  |  |
| Oesophageal cancer | T2D + OSA | 42,175 | 41 | 1.025 | (0.665, 1.580) |
|  | T2D | 42,189 | 41 |  |  |
| Endometrial cancer | T2D + OSA | 41,950 | 87 | 0.866 | (0.651, 1.152) |
|  | T2D | 41,969 | 103 |  |  |
| **All-cause mortality, dementia and liver outcomes** | **Cohorts** | **Sample size** ^†^ | **Outcome** | **Hazard ratio** | **95% CI** |
| All-cause mortality | T2D + OSA | 42,242 | 4,133 | 1.165 | (1.114, 1.218) |
|  | T2D | 42,242 | 3,641 |  |  |
| Dementia | T2D + OSA | 41,275 | 769 | 1.153 | (1.040, 1.278) |
|  | T2D | 41,452 | 688 |  |  |
| Metabolic dysfunction-associated steatotic liver disease | T2D + OSA | 38,944 | 2,188 | 1.248 | (1.173, 1.328) |
|  | T2D | 39,580 | 1,847 |  |  |
| Metabolic dysfunction-associated steatohepatitis | T2D + OSA | 41,466 | 560 | 1.521 | (1.336, 1.733) |
|  | T2D | 41,752 | 381 |  |  |

T2D: Type 2 diabetes. OSA: Obstructive sleep apnoea. CKD: Chronic kidney disease. ^†^number of participants
